# First record of the genus *Patchiella* (Hemiptera, Aphididae) on *Tilia mandshurica* in South Korea

**DOI:** 10.3897/BDJ.14.e185488

**Published:** 2026-03-26

**Authors:** Minho Lee, Mariusz Kanturski, Natalia Kaszyca-Taszakowska, Seunghwan Lee

**Affiliations:** 1 Seoul National University, Seoul, Republic of Korea Seoul National University Seoul Republic of Korea; 2 University of Silesia in Katowice, Katowice, Poland University of Silesia in Katowice Katowice Poland

**Keywords:** aphids, pest, new record, morphology, biodiversity

## Abstract

**Background:**

The aphid genus *Patchiella* Tullgren, 1925 (Hemiptera, Aphididae, Pemphiginae, Pemphigini) is known for forming characteristic “leaf nests” on *Tilia* species and alternating to Araceae as secondary hosts. Although *Patchiella kolokasia* has been reported from China and Japan, it has never been recorded from South Korea.

**New information:**

*Patchiella kolokasia* (Qiao & Zhang, 1999) is recorded for the first time from South Korea, collected on *Tilia mandshurica* Rupr. et Maxim. (Malvaceae) in Gyeonggi-do, South Korea. Detailed morphological observations of fundatrices are provided. This discovery expands the known distribution of *P. kolokasia* to the Korean Peninsula and represents the first record of the genus *Patchiella* in South Korea.

## Introduction

Honey-producing plants play a pivotal role in supporting pollinator activity and their availability directly influences both the productivity and stability of the apiculture industry (Klein et al. 2007, Potts et al. 2010, Vaudo et al. 2015). In South Korea, the beekeeping industry generates substantial economic value, with honey production accounting for approximately 53.7% of the total production value, corresponding to 1,228 billion KRW out of a total of 2,288 billion KRW (Lee et al. 2019, Kim et al. 2023). However, this strong dependence on honey production renders the industry particularly vulnerable to climate change and outbreaks of pests and diseases, as declines in honey yield directly translate into income losses for beekeeping households (Lee et al. 2019, Kim et al. 2023). *Tilia mandshurica* Rupr. et Maxim. (Malvaceae) is one of the major honey plants in South Korea and is widely distributed across both urban and forest habitats (Lee 1998, Choi et al. 2021); nevertheless, research on sap-feeding aphids affecting this species remains remarkably limited, despite their potential significance for plant protection, pest management and the long-term sustainability of pollinator-supporting vegetation.

The genus *Patchiella* Tullgren, 1925 (Hemiptera, Aphididae, Pemphiginae, Pemphigini) comprises host-alternating woolly aphids predominantly distributed across the Palaearctic Region (Kurosu et al. 2022). Currently, two valid species and one subspecies are recognised (Kaltenbach 1843, Pashtshenko 1984, Pashtshenko 1988, Qiao and Zhang 1999, Favret 2026): *Patchiella reaumuri reaumuri* (Kaltenbach, 1843), *P. reaumuri orientalis* Pashtshenko, 1984 and *P. kolokasia* (Qiao & Zhang, 1999). Species of this genus exhibit a complex heteroecious life cycle, in which fundatrices produce conspicuous “leaf-nest” pseudogalls on *Tilia* spp. (Malvaceae) during spring, followed by the host alternation of alate morphs migrating to the root systems of Araceae plants, including *Colocasia* and *Arum* spp., where they complete the subterranean feeding phase (Roberti 1939, Börner and Heinze 1957, Stroyan 1979, Dixon and Thieme 2007, Firake et al. 2022, Kurosu et al. 2022, Favret C & Aphid Taxon Community, eds. 2026).

*Patchiella kolokasia* was originally described from China by Qiao and Zhang (1999) and was later re-assigned to *Patchiella* by Kurosu et al. (2022). This species exhibits several diagnostic characteristics, including an unbranched media vein on the fore-wing and distinctly asymmetric claws in the first-instar nymphs. A notable behavioural trait reported by Kurosu et al. (2022) is the near-complete coverage of the fundatrix’s body by its progeny within the leaf nest Kurosu et al. (2022). To date, *Patchiella kolokasia* has been recorded only from China and Japan (Qiao and Zhang 1999, Zhang et al. 1999, Kurosu et al. 2022) and no species of *Patchiella* have previously been reported from South Korea. During surveys of early spring nectar-producing plants, an unusual leaf-nest colony was discovered on *Tilia mandshurica* Rupr. et Maxim. (Malvaceae) in Gyeonggi-do, South Korea and subsequent morphological examination confirmed the identity of the species as *P. kolokasia* (Qiao & Zhang, 1999). In China and Japan, this species has also been observed feeding on taro (*Colocasia esculenta*), an economically important root crop, as a secondary host, raising concerns regarding its potential role as an agricultural and horticultural pest (Qiao and Zhang 1999, Zhang et al. 1999, Kurosu et al. 2022). The detection of *Patchiella kolokasia* on a key native honey plant in South Korea represents not only a significant biogeographical record, but also a critical early warning for plant protection, emphasising the necessity of proactive surveillance, continuous monitoring and systematic risk assessment of host-alternating aphids that pose emerging threats to pollinator-supporting vegetation and economically important crops.

## Materials and methods

**Figure 1.**
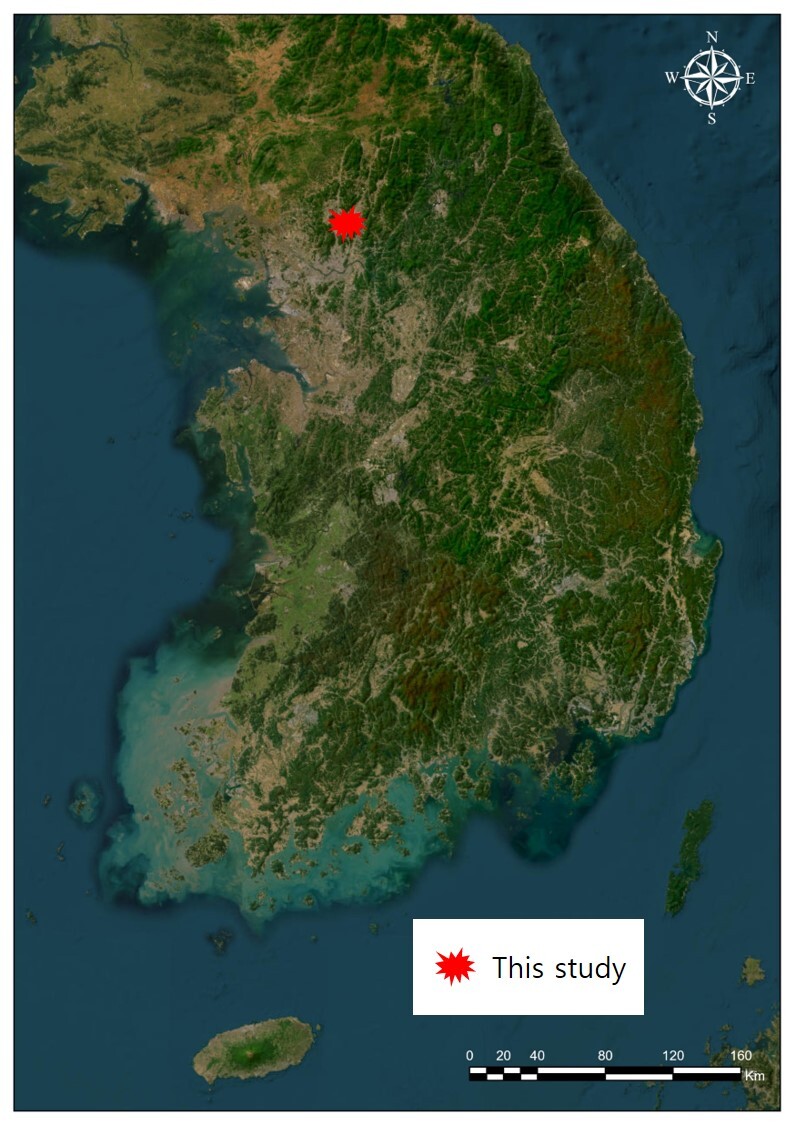
Distribution of *Patchiella kolokasia* (Qiao & Zhang, 1999) in South Korea.

Aphid colonies were collected from characteristic “leaf-nest” pseudogalls on *Tilia mandshurica* Rupr. et Maxim. (Malvaceae) in Gyeonggi-do, South Korea on 3 May 2021 (Fig. 1). The aphid samples were preserved in 90% ethanol and slide-mounted specimens were prepared in Canada balsam following the method described by Favret C & Aphid Taxon Community, eds. (2026). Measurements and digital images were taken using Leica DMC 5400 (Leica Z16 APO) and Leica DM 4000B (Active Measure version 3.0.3; Mitani Co. Ltd., Japan) cameras. Abbreviations used for descriptions are as follows: ANT, antennae; ANT III, ANT IV; antennal segment III, IV, respectively; GP, genital plate; HT I, first segment of hind tarsus; HT II, second segment of hind tarsus; URS, ultimate rostral segment (segment IV+V); ABD TERG I-VIII, abdominal tergites I–VIII; BL, body length; TIBIAE III, hind tibiae. All specimens and slide vouchers were deposited in the College for Agriculture and Life Sciences, Seoul National University Seoul, South Korea (SNU).

## Taxon treatments

### Patchiella kolokasia

(Qiao & Zhang, 1999)

4B7C36FE-8FE5-523A-BB09-B3AFC80081FD

*Gharesia kolokasia* Qiao & Zhang, 1999: 172. - Qiao and Zhang (1999)
*Patchiella kolokasia* (Qiao & Zhang, 1999) in Kurosu, Aoki & Arai, 2022: 314. - Kurosu et al. (2022)

#### Materials

- **Type status:** Other material. **Occurrence:** catalogNumber: PaLMH1; recordedBy: Minho Lee; individualCount: 1; sex: female; lifeStage: Fundatrix; occurrenceID: 3A5466C5-2F98-5009-9573-C761B256CE10; **Taxon:** scientificName: Patchiella kolokasia; order: Hemiptera; family: Aphididae; genus: Patchiella ; scientificNameAuthorship: (Qiao & Zhang, 1999); **Location:** country: South Korea; stateProvince: Gyeonggi-do; locality: Soheul-eup, Pocheon-si; locationRemarks: Soheul-eup, Pocheon-si, Gyeonggi-do, South Korea (GPS: 37.7550, 127.1658); verbatimCoordinates: 37.7550, 127.1658; decimalLatitude: 37.755; decimalLongitude: 127.1658; **Identification:** identifiedBy: https://orcid.org/0000-0002-4012-8928; dateIdentified: 2021; **Event:** eventDate: 03-May-2021; **Record Level:** language: en; collectionCode: Insects; basisOfRecord: PreservedSpecimen
- **Type status:** Other material. **Occurrence:** catalogNumber: PaLMH2; recordedBy: Minho Lee; individualCount: 1; sex: female; lifeStage: Fundatrix; occurrenceID: 1FED3273-EA03-5332-B067-3097F5A64CC6; **Taxon:** scientificName: Patchiella kolokasia; order: Hemiptera; family: Aphididae; genus: Patchiella ; scientificNameAuthorship: (Qiao & Zhang, 1999); **Location:** country: South Korea; stateProvince: Gyeonggi-do; locality: Soheul-eup, Pocheon-si; locationRemarks: Soheul-eup, Pocheon-si, Gyeonggi-do, South Korea (GPS: 37.7550, 127.1658); verbatimCoordinates: 37.7550, 127.1658; decimalLatitude: 37.755; decimalLongitude: 127.1658; **Identification:** identifiedBy: https://orcid.org/0000-0002-4012-8928; dateIdentified: 2021; **Event:** eventDate: 03-May-2021; **Record Level:** language: en; collectionCode: Insects; basisOfRecord: PreservedSpecimen
- **Type status:** Other material. **Occurrence:** catalogNumber: PaLMH3; recordedBy: Minho Lee; individualCount: 1; sex: female; lifeStage: Fundatrix; occurrenceID: AA881EDC-7349-5218-AA87-541B580946EE; **Taxon:** scientificName: Patchiella kolokasia; order: Hemiptera; family: Aphididae; genus: Patchiella ; scientificNameAuthorship: (Qiao & Zhang, 1999); **Location:** country: South Korea; stateProvince: Gyeonggi-do; locality: Soheul-eup, Pocheon-si; locationRemarks: Soheul-eup, Pocheon-si, Gyeonggi-do, South Korea (GPS: 37.7550, 127.1658); verbatimCoordinates: 37.7550, 127.1658; decimalLatitude: 37.755; decimalLongitude: 127.1658; **Identification:** identifiedBy: https://orcid.org/0000-0002-4012-8928; dateIdentified: 2021; **Event:** eventDate: 03-May-2021; **Record Level:** language: en; collectionCode: Insects; basisOfRecord: PreservedSpecimen

#### Diagnosis

Fundatrix.

**Figure 2.**
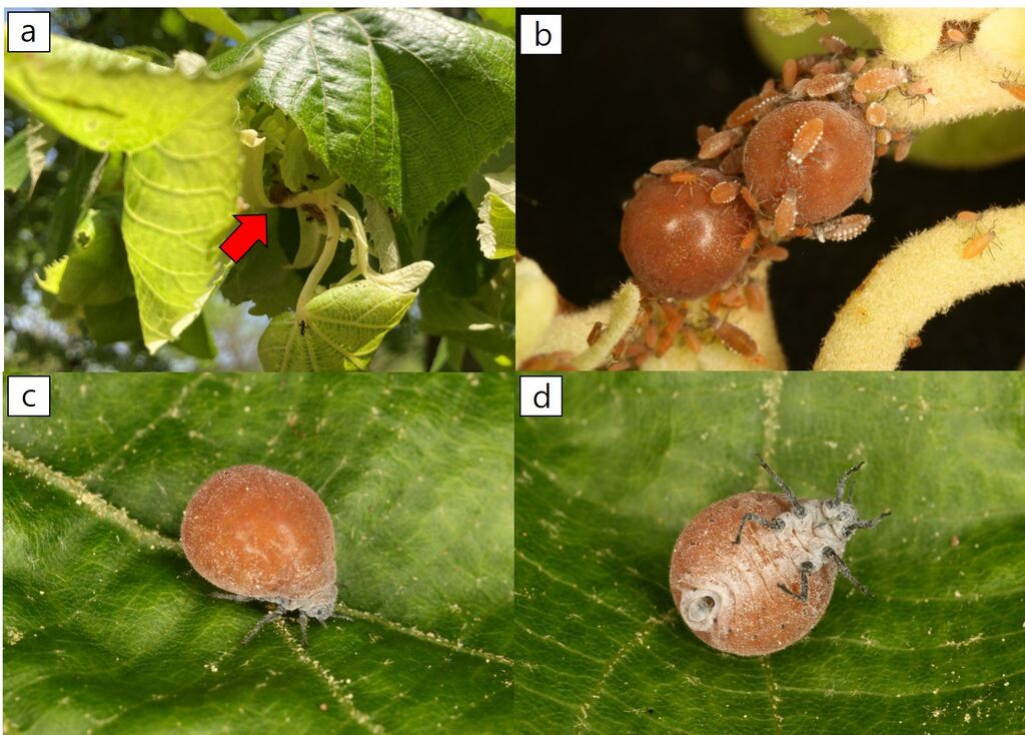
*Patchiella kolokasia* (Qiao & Zhang, 1999) in life: **a** colonies forming “leaf-nest” pseudogalls on *Tilia mandshurica*; **b** colony of fundatrices with nymphs; **c** dorsal view of a fundatrix; **d** ventral view of a fundatrix.

**Figure 3.**
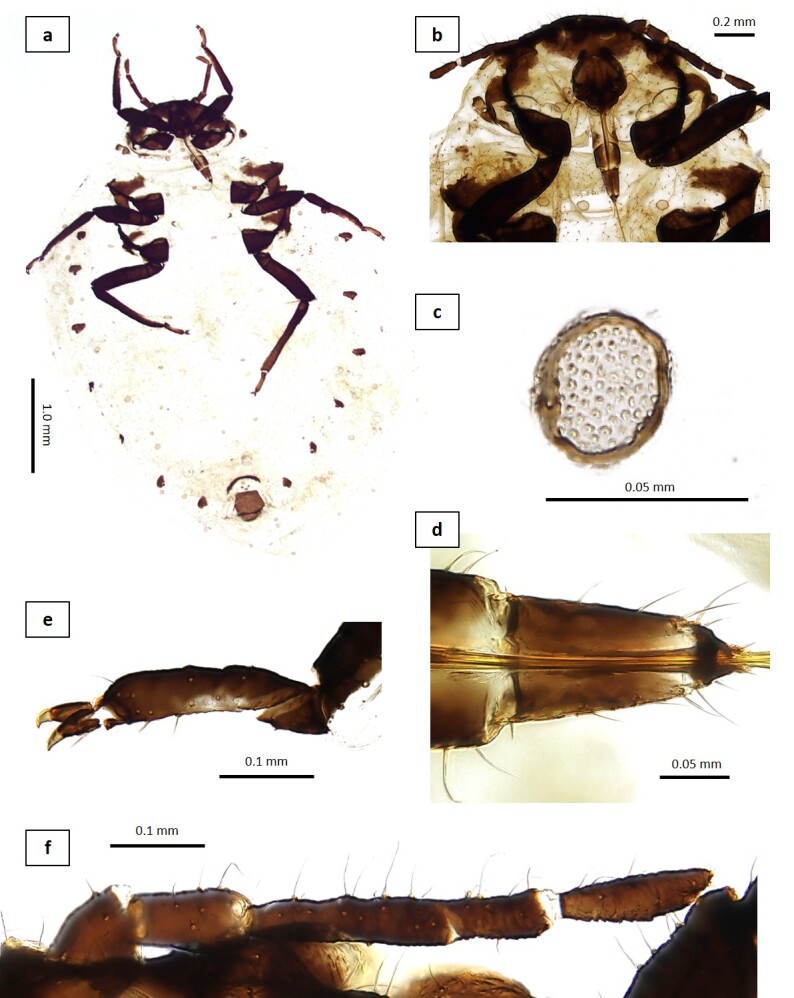
*Patchiella kolokasia* (Qiao & Zhang, 1999): specimen's catalogue number (PkLMH1-3), fundatrix: **a** body (PkLMH3); **b** head with thorax (PkLMH1); **c** wax plate (PkLMH3); **d** URS (PkLMH1); **e** HT II (PkLMH2); **f** ANT (PkLMH1).

**Colour. In life**: head and thorax dark brown, white powdered with wax. ABD TERG I–VIII orangish-brown or brown. Leg and ANT dark brown (Figs 2, 3).

**Morphometric characters (Table 1**): body globular, ANT 0.13–0.15 × BL. Antenna 4-segmented, ANT IV 0.45–0.52 × ANT III. URS 0.52–0.61 × ANT III, 1.06–1.19 × ANT IV and 0.74–0.86 × HT II with 6–7 fine accessory setae. HT I 0.30–0.35 × HT II. GP U-shaped with 32–39 fine setae. Siphunculi absent.

#### Remarks

This species was first discovered on *Tilia mandshurica* in South Korea. The diagnostic characteristics of the Korean specimens, including the presence of dorsal wax plates on the head, a four-segmented antenna, the number of setae on the basal part of ANT IV, the length and number of secondary setae on URS and the relative lengths of the femorotrochanter and HT II, are consistent with the fundatrix description of the Japanese population provided by Kurosu et al. (2022). Furthermore, the number and arrangement of setae on both the cauda and the anal plate are consistent with those previously reported. In contrast to the Japanese specimens, the Korean individuals possess distinct wax plates on the thoracic and abdominal tergites.

#### Distribution

Previously known from Japan and China. Newly recorded from South Korea.

## Identification Keys

### Key to the fundatrix of *Patchiella* species on *Tilia* spp. (Pashtshenko (1984); Kurosu et al. (2022); Favret C & Aphid Taxon Community, eds. (2026)).

**Table:** 

1	Colour in life: Light brownish-pink or yellowish-brown to greenish. ANT relatively long, more than 0.16 × BL, URS relatively short, less than 0.40 × ANT III	2
–	Colour in life: Orangish-brown or brown. ANT short, 0.13–0.15 × BL, URS long, 0.52–0.61 × ANT III	*Patchiella kolokasia* (Qiao & Zhang, 1999)
2	Colour in life: Light brownish-pink. URS 0.32-0.36 × ANT III, URS 0.82-1.07 × HT II	*Patchiella reaumuri orientalis* Pashtshenko, 1984
–	Colour in life: Yellowish-brown to greenish. URS 0.31-0.32 × ANT III, URS 0.76-0.82 × HT II	*Patchiella reaumuri reaumuri* (Kaltenbach, 1843)

## Discussion

This study documents the first confirmed record of the genus *Patchiella* in South Korea, identified as *Patchiella kolokasia* on the native honey plant *Tilia mandshurica*. The diagnostic characters of the Korean specimens, including the four-segmented antennae, the number of setae on the basal part of ANT IV, the proportional length of URS relative to ANT III, ANT IV and HT II and the number of setae on both the cauda and the anal plate, were fully consistent with the description of the fundatrix population from Japan (Kurosu et al. 2022). These results strongly support the identity of the Korean specimens as *P. kolokasia* and indicate an expansion of the known distribution of this species to the Korean Peninsula.

A notable difference was observed in the presence of distinct wax plates on the thoracic and abdominal tergites of the Korean specimens, a character not previously reported from Japanese populations (Kurosu et al. 2022). This feature may reflect intraspecific variation or environmentally influenced morphological plasticity associated with local conditions. Additional material representing multiple morphs and seasons, combined with molecular data, will be necessary to clarify whether this character is geographically structured or reflects phenotypic variability within the species.

Beyond its taxonomic significance, the detection of *P. kolokasia* on *T. mandshurica* has important implications for plant protection. *Tilia mandshurica* is a key native honey plant in South Korea, contributing substantially to pollinator support and apicultural productivity. Damage to this host may, therefore, have cascading effects on pollinator resources and ecosystem services. In addition, *P. kolokasia* is a heteroecious aphid known to alternate from *Tilia* species to secondary hosts within the Araceae, including taro (*Colocasia esculenta*), an economically important agricultural crop in East Asia (Qiao and Zhang 1999, Zhang et al. 1999, Kurosu et al. 2022). This host-alternating life cycle increases the potential risk of pest spread across both natural and agricultural systems.

Host-alternating aphids are of particular concern in plant protection because population build-up on the primary host can facilitate subsequent colonisation of secondary hosts. In the case of *P. kolokasia*, fundatrices induce conspicuous leaf-nest pseudogalls on *Tilia*, resulting in leaf rolling and physical deformation of host tissues. Such visible damage on the primary host provides an opportunity for early detection and monitoring before alate morphs migrate to secondary hosts. Proactive surveillance of leaf-nest formation and leaf-rolling symptoms on *Tilia* spp. may, therefore, serve as an effective early-warning indicator to prevent further spread and reduce the risk of damage to both primary honey plants and secondary agricultural crops such as taro.

Accordingly, protecting both pollinator-supporting honey plants and economically important crops requires an integrated monitoring strategy that considers the full heteroecious life cycle of aphids, such as *P. kolokasia* (Kurosu et al. 2022). Early detection on the primary host, followed by targeted surveys of potential secondary hosts, may help limit population expansion and mitigate subsequent impacts. Continuous monitoring of *T. mandshurica* populations, particularly during early spring when fundatrices establish leaf nests, will be essential for assessing the establishment status and spread potential of this species in South Korea. Such efforts will contribute to risk assessment and informed management strategies for host-alternating aphids that pose emerging threats to both apicultural resources and agricultural systems.

## Supplementary Material

XML Treatment for Patchiella kolokasia

## Figures and Tables

**Table 1.** Biometric measurements of *Patchiella kolokasia* (Qiao & Zhang, 1999) (min.–max.) mean length (mm).

character	fundatrix (n = 3)		
BL	(4.53-4.92) 4.67		
ANT I	(0.06-0.08) 0.07		
ANT II	(0.10-0.11) 0.11		
ANT III	(0.31-0.33) 0.32		
ANT IV	(0.15-0.17) 0.16		
URS	(0.17-0.19) 0.18		
Femorotrochanter	(0.80-0.83) 0.82		
TIBIAE III	(0.91-0.97) 0.94		
HT I	(0.07-0.08) 0.07		
HT II	(0.22-0.23) 0.22		
Wax plate	Number of spinal wax plate	Number of marginal wax plate	Total wax plate
Head	2	4	6
Prothorax	4	0	4
Mesothorax	2	4	6
Metathorax	2	4	6
ABD TERG I	2	4	6
ABD TERG II	2	4	6
ABD TERG III	2	4	6
ABD TERG IV	2	4	6
ABD TERG V	2	2	4
ABD TERG VI	2	2	4
ABD TERG VII	2	2	4
